# AMPK and PKA interaction in the regulation of survival of liver cancer cells subjected to glucose starvation

**DOI:** 10.18632/oncotarget.7404

**Published:** 2016-02-15

**Authors:** Anabela C. Ferretti, Facundo M. Tonucci, Florencia Hidalgo, Evangelina Almada, Maria C. Larocca, Cristián Favre

**Affiliations:** ^1^ Institute of Experimental Physiology, CONICET, School of Biochemical Sciences, University of Rosario, Rosario, Argentina

**Keywords:** AMPK, apoptosis, cancer cell killing, hepatocellular carcinoma, signaling

## Abstract

The signaling pathways that govern survival response in hepatic cancer cells subjected to nutritional restriction have not been clarified yet. In this study we showed that liver cancer cells undergoing glucose deprivation both arrested in G0/G1 and died mainly by apoptosis. Treatment with the AMPK activator AICAR phenocopied the effect of glucose deprivation on cell survival, whereas AMPK silencing in HepG2/C3A, HuH-7 or SK-Hep-1 cells blocked the cell cycle arrest and the increase in apoptotic death induced by glucose starvation. Both AMPK and PKA were promptly activated after glucose withdrawal. PKA signaling had a dual role during glucose starvation: whereas it elicited an early decreased in cell viability, it later improved this parameter. We detected AMPK phosphorylation (AMPKα(Ser173)) by PKA, which was increased in glucose starved cells and was associated with diminution of AMPK activation. To better explore this inhibitory effect, we constructed a hepatocarcinoma derived cell line which stably expressed an AMPK mutant lacking that PKA phosphorylation site: AMPKα1(S173C). Expression of this mutant significantly decreased viability in cells undergoing glucose starvation. Furthermore, after 36 h of glucose deprivation, the index of AMPKα1(S173C) apoptotic cells doubled the apoptotic index observed in control cells. Two main remarks arise: 1. AMPK is the central signaling kinase in the scenario of cell cycle arrest and death induced by glucose starvation in hepatic cancer cells; 2. PKA phosphorylation of Ser173 comes out as a strong control point that limits the antitumor effects of AMPK in this situation.

## INTRODUCTION

Among liver cancers, hepatocellular carcinoma (HCC), which proceeds from the transformation of the hepatocytes and represents the 90% of cancer from liver cells, ranks as one of the three highest lethal cancers. The eligible chemotherapy, sorafenib [1], is highly toxic and poorly effective; therefore alternative or complementary strategies for treatments are required [2]. Hepatocarcinoma derived cells are a good model to explore the metabolic hallmarks and mechanisms of death of these tumor cells. Specially, HCC derived HepG2 and HuH-7 cell lines have been studied for decades. HCC cells show an extremely elevated glycolytic flux, which has been very well characterized by metabolomics analysis in the case of HepG2/C3A cells [3]. The response of HCC cells to glucose starvation has not been deeply studied; it is nevertheless a promising field for detecting putative therapeutic targets. Glucose deprivation induces cell death in hepatocarcinoma derived HepG2 cells, even though neither the type of death nor the underlying mechanism is clear. In fact, two different reports indicate that cells die by apoptosis [4, 5], but another one proposes caspase 8 dependent necrosis as the main cause of cell death [6].

In previous studies, we have shown the existence of a mitochondrial cAMP-protein kinase A (PKA) axis in normal hepatocytes, which signals the response to the lack of glucose by increasing ROS production and inducing apoptotic activation [7]. Interestingly, this PKA signaling pathway that decreases cell survival during stress induced by low glucose is likely conserved from yeast [8]. Up regulation of cAMP-PKA pathway has also been suggested to inhibit cell cycle progression and survival in HCC derived cell lines [9]. However, a direct participation of PKA signaling in the growth control of HCC cells has not been confirmed except for a recent work which shows that PKA activates a tumor suppressor [10].

On the other hand, AMP activated kinase (AMPK) is a key signaling kinase in the response to energetic stress, which regulates glucose and lipid metabolism, as well as cell survival [11]. During the last years, several studies performed in HCC derived cells, show that activation of AMPK by different activators entails either cell cycle arrest, as in the case of AICAR and metformin [12, 13], or cell death, as in the case of cannabinoids and berberine [14, 15]. Therefore, the metabolic context and the mode of activation of this kinase seem to condition its effects on cell proliferation and/or viability. AMPK is a heterotrimeric complex in which the catalytic subunit AMPKα possesses a Thr residue (T172) that is phosphorylated in the active form of this kinase [11]. Phosphorylation by PKA of the contiguous Ser residue (S173) impedes the normal phosphorylation of T172, thus decreasing AMPKα activation in adipocytes [16], where counteraction of AMPK and PKA during lipolysis is well described [17].

Collectively, all the evidence supports the hypothesis that survival response in HCC cells undergoing glucose starvation is the outcome of a signaling network commanded by AMPK and PKA. In the present study we investigated cell cycle progression and viability of hepatic cancer derived cells exposed to this condition, and demonstrated the involvement of each kinase in the control of survival. Counteraction on AMPK by PKA was also analyzed and confirmed in AMPKα1(S173) mutant cells. Moreover, sensitization to glucose restriction was achieved in HCC cells expressing S173C unphosphorylable AMPKα1.

## RESULTS

### Rapid loss of survival in HCC cells undergoing glucose restriction or AMPK activation is associated to cell cycle arrest and apoptotic/necrotic death

**Figure 1:**
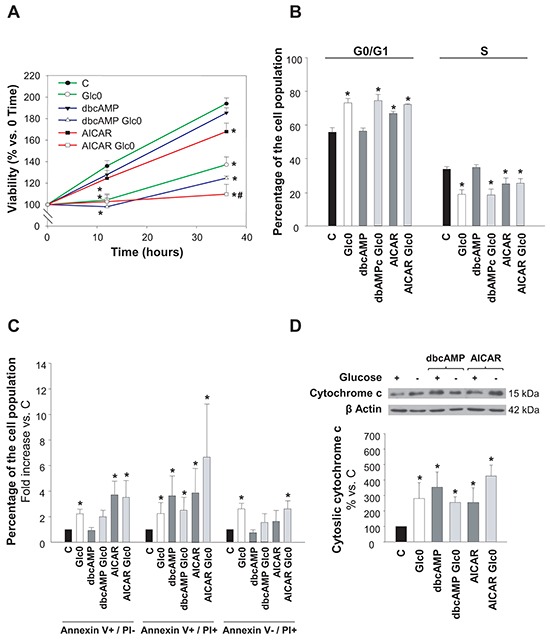
Hepatocarcinoma derived cells survival during glucose deprivation C3A cells were incubated with 4.5 g/L glucose DMEM (C) or with no-glucose DMEM (Glc0), in the absence or presence of 100 μM dbcAMP (dbcAMP) or 1 mM AICAR (AICAR). **A.** Cells attached to microplates were cultured for 0, 12 and 36 h. MTT assay was performed as described in *Materials and Methods*. Results are presented as the percentage of the absorbance at 0 h. **B.** After 36 h, cells were fixed with cold 70% ethanol, stained with propidium iodide, and analyzed by flow cytometry. Bars show the percentage of cells in G0/G1 and S phases of the cell cycle. **C.** After 36 h, cells were stained with Annexin V-FITC and propidium iodide (PI) and the percentages of apoptotic (Annexin V+) and dead (PI+) cells were determined by flow cytometry analysis. Bar charts represent increases in the percentages of cells undergoing early apoptosis (Annexin V+/PI−), late apoptosis (Annexin V+/PI+) and primary necrosis (Annexin V-/PI+) relative to controls. The composition of control cell population was (mean values): early apoptotic cells, 3%; late apoptotic cells, 4%; primary necrotic cells, 7%. **D.** After 36 h of treatment, cytosolic fractions were obtained and analyzed by western blotting to determine protein levels of cytochrome c. β Actin was used as loading control. Values represent the mean ± SEM of 6 (A), 3 (B, C) or 4 (D) experiments. *P<0.05 versus C. ^#^*P*<0.05 versus Glc0.

It has been described that glucose deprivation induces cell death in HCC derived cells [4–6], however the studies on this subject are scarce and the underlying mechanism is unclear. First, we characterized the effect of glucose restriction on cell viability along time in normal, AICAR or dibutyryl-cAMP treated HepG2/C3A cells. We showed that lack of glucose rapidly led to decreased cell viability, which was already significant after 12 h of starvation (Figure 1A). Progressive decrease in viability after glucose withdrawal was observed also in the HCC derived HuH-7 cells (−23% at 36 h). In addition, liver adenocarcinoma derived SK-Hep-1 cells showed similar results (−30% at 36 h), thus indicating that this behavior was not exclusive for HCC cells (data not shown).

Activation of AMPK by AICAR also decreased cell viability, and significantly enhanced the loss in cell viability induced by glucose restriction. Instead, activation of PKA by dibutyryl-cAMP (dbcAMP) did not produce any significant change in the number of viable cells neither in glucose fed nor in fasted cultures (Figure 1A).

We next determined the putative changes in cell cycle progression and in the induction of cell death. We observed that the population of cells in G0/G1 was significantly increased by the lack of glucose, as well as by AMPK activation (+31% and +20% at 36 h, respectively). No significant effects were registered in cell cycle progression with PKA activation by dbcAMP at 36 h (Figure 1B). Cytometric Annexin V-PI assay at this time demonstrated that both apoptotic and necrotic death were increased almost twice in glucose deprived cells, and that the treatments with AICAR or dbcAMP also led to significant augment in the fraction of apoptotic cells without inducing primary necrotic death (Figure 1C). Apoptotic activation was also confirmed by detection of cytochrome c release to cytosol (Figure 1D).

### AMPK and PKA are promptly activated during glucose starvation

**Figure 2:**
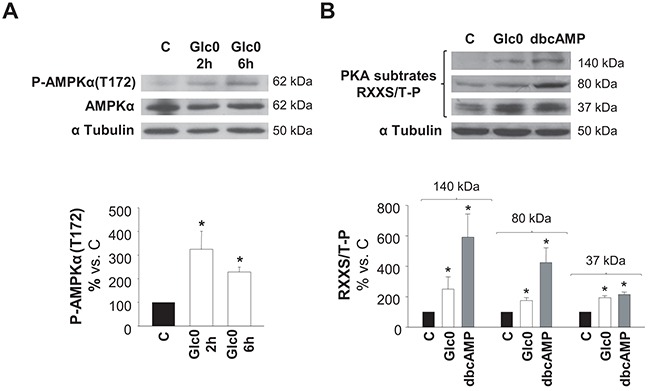
AMPK and PKA activation during glucose restriction C3A cells were incubated with 4.5 g/L glucose DMEM (C) or with no-glucose DMEM (Glc0). Cell lysates were obtained and analyzed by western blotting. **A.** Protein levels of P-AMPKα(T172) and AMPKα were detected at 2 and 6 h of glucose deprivation. **B.** Phosphorylated PKA substrates (RXXS/T-P residues) were detected after 2 h of treatments. Cells incubated with 100 μM dbcAMP (dbcAMP) were used as positive control of PKA phosphorylation. Bars represent band densities of PKA substrates weighted 37, 80 and 140 kDa. α Tubulin was used as loading control. Values represent the mean ± SEM of 3 experiments. *P<0.05 versus C.

We determined whether AMPK and/or PKA kinases were activated by glucose deprivation. Detection of phospho-AMPKα (Thr172) by a specific antibody and detection of Ser/Thr residues phosphorylated by PKA by an antibody recognizing phosphorylated consensus sites were performed in control and glucose deprived C3A cells at different times. Both increased levels of P-AMPKα (Thr172) and increased phosphorylation of PKA substrates (37, 80 and 140 kDa) at 2 h demonstrated early activation of these kinases during lack of glucose (Figure 2).

All together, data illustrated in Figures 1 and 2 indicated that glucose deprivation both impeded progression of HepG2/C3A cells into S phase and induced their death not only by apoptosis but also by necrosis, what explained the gradual decrease observed in the number of viable cells observed in that condition. Besides, independent activation of AMPK or PKA entailed apoptosis, which, in the case of AMPK activation, occurred in the presence of cell cycle arrest. Moreover, AMPK and PKA kinases showed simultaneous activation after glucose withdrawal. This first round evidence raised both kinases as good candidates to control the survival response to glucose restriction in HCC cells.

### Silencing the expression of AMPK entails partial rescue of cell cycle arrest and death after glucose withdrawal

**Figure 3:**
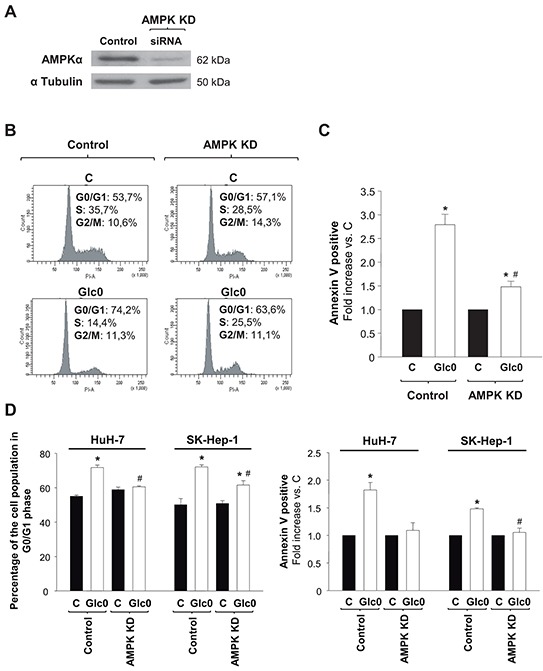
Effects of AMPK knock down on cell cycle progression and cell death during glucose deprivation in liver cancer cells C3A (A, B, C) HuH-7 and SK-Hep-1 (D) cells were transfected with a siRNA specifically targeted to AMPKα1 mRNA (AMPK KD), or with a siRNA control (Control), and allowed to grow for 48 hours. **A.** AMPKα expression was analyzed by western blot, in Control and AMPK KD cells. α Tubulin was used as loading control. In panels B, C and D control and AMPK KD cells were incubated with 4.5 g/L glucose DMEM (C) or with no-glucose DMEM (Glc0) during 24 h. **B.** Cells were fixed, stained with propidium iodide, and analyzed by flow cytometry. The figure shows typical outputs of the cytometric analysis and percentages of C3A cells in the different phases of the cell cycle, an experiment representative of 3 independent experiments. **C.** C3A cells were stained with Annexin V-FITC and propidium iodide (PI) and the percentages of apoptotic cells were determined by flow cytometry analysis. Bar charts represent increase in the percentages of cells undergoing apoptosis (Annexin V positive) relative to controls (C). Control apoptotic cells (mean value): 8%. Values represent the mean ± SEM of 3 experiments. *P<0.05 versus C. ^#^*P*<0.05 versus Glc0. **D.** Bar charts (left) show the percentage of cells in G0/G1 phase of the cell cycle in Control and AMPK KD HuH-7 and SK-Hep-1 cells treated as in B. Bar charts (right) illustrate increases in apoptotic cells in Glc0 vs C in Control and AMPK KD HuH-7 and SK-Hep-1 cells treated as in C. Values represent the mean ± SEM of 3 experiments. *P<0.05 versus C. ^#^*P*<0.05 versus Glc0.

In order to determine the specific contribution of AMPK signaling to cell cycle progression and death during glucose deprivation in liver cancer cells, we reduced the expression of the most abundant isoform of the catalytic subunit of AMPK, AMPKα1 [18, 19], by RNA interference in HepG2/C3A, HuH-7, and SK-Hep-1. Cells transfected with a siRNA specifically targeting AMPKα1 (AMPK KD) showed an average 80% decrease in AMPKα expression (Figure 3A). The decrease in AMPKα expression partially prevented the increase in the G0/G1 population induced by 24 h-glucose deprivation in the paradigmatic HCC derived cells HepG2/C3A (control +38%, AMPK KD +11%, *P*<0.05) (Figure 3B). Cell death assays showed that the number of total apoptotic cells was almost three fold increased by 24 h-glucose deprivation in control, but only 50% increased in AMPK KD HepG2/C3A cells subjected to the same condition (*P*<0.05) (Figure 3C). Similar tendencies were showed in HuH-7 cells and in non-epithelial SK-Hep-1 cells as well (Figure 3D). Altogether these data suggested a key role for AMPK activity in limiting survival in glucose deprived hepatic cancer cells.

### Effect of PKA inhibition on cell survival during glucose deprivation

**Figure 4:**
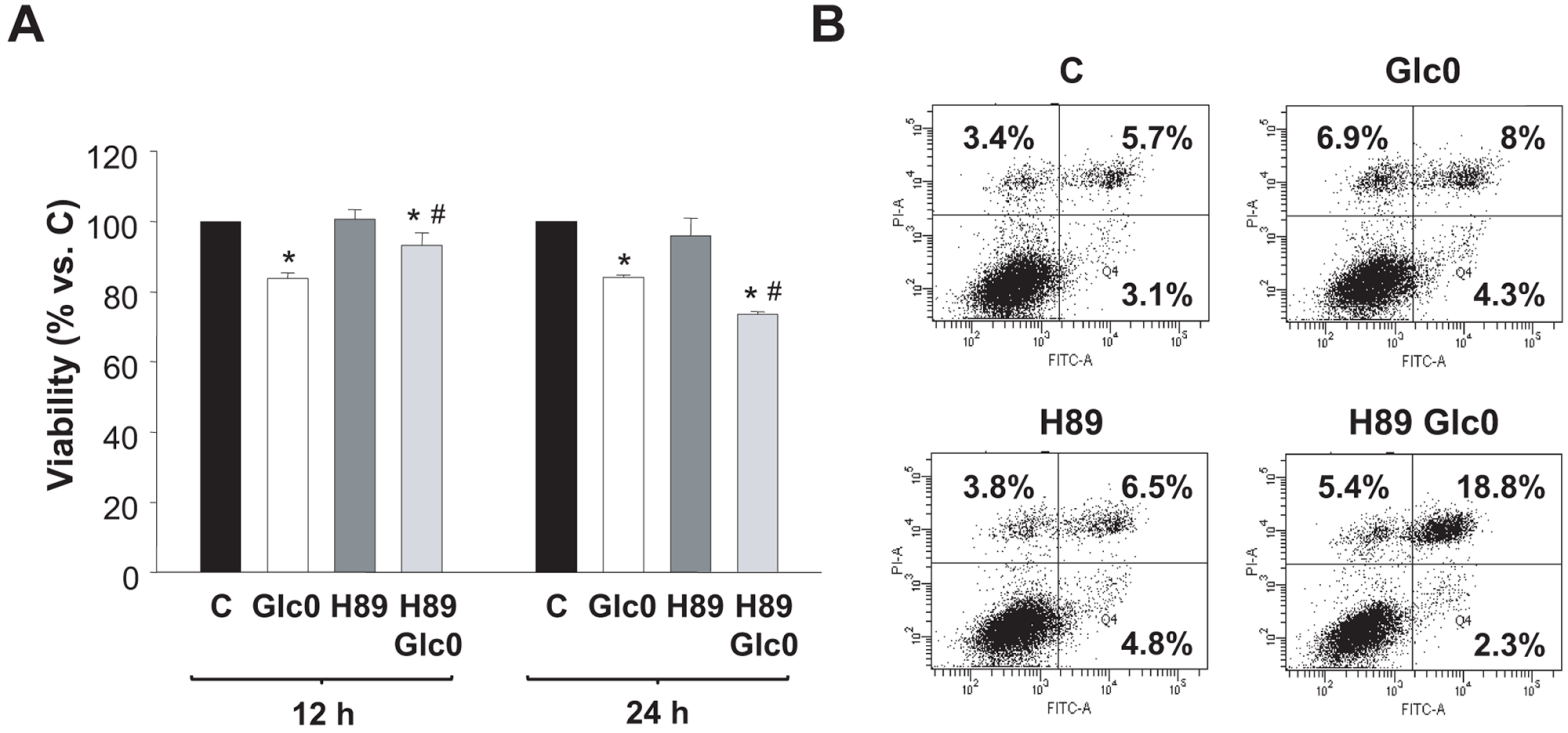
Effects of PKA inhibition on cell viability during glucose deprivation C3A cells were incubated with 4.5 g/L glucose DMEM (C) or with no-glucose DMEM (Glc0), in the absence or presence of 5 μM H89 (H89). **A.** Cells attached to microplates were cultured for 12 and 24 h. MTT assay was performed as described in *Materials and Methods*. Results are presented as the percentage of control cells absorbance at each time. Values represent the mean ± SEM of 5 experiments. *P<0.05 versus C. ^#^*P*<0.05 versus Glc0. **B.** After 24 h of treatment, cells were stained with Annexin V-FITC and propidium iodide (PI) and analyzed by flow cytometry. The figure shows outputs of the cytometric analyses and percentages of cells undergoing early apoptosis (bottom right), late apoptosis (upper right) and primary necrosis (upper left) of an experiment representative of 3 independent experiments.

To determine the involvement of PKA activation in the survival response to glucose withdrawal in C3A cells, we studied the effect of the PKA inhibitor H89 at different times. At 12 h of culture, H89 partially blocked the loss of viability provoked by glucose withdrawal, while, conversely, at 24 h the inhibitor significantly potentiated the effect of glucose deprivation on cell viability (Figure 4A). These results suggested a dual role of PKA during glucose starvation in C3A cells. The initial antitumorigenic action was in accordance with the existence of a cAMP/PKA axis that signals apoptotic activation that we observed in normal hepatocytes after glucose deprivation, which is associated to increased production of radical oxygen species (ROS) [7]. In fact, an early accumulation of ROS induced by PKA activation is also detected in C3A cells undergoing glucose starvation (Ferretti et al. unpublished). Regarding the pro-survival role of PKA at later times, we discarded any effect on cell cycle: no significant changes in the populations were detected in glucose-fed and fasted C3A cells in the presence of H89 (data not shown). This indicated that, after 24 h, cell death induced by glucose deprivation would be directly or indirectly prevented by PKA activation. To analyze the effect of PKA inhibition on apoptosis, we determined Annexin V staining by flow citometry. Our results showed that the percentage of H89 treated cells subjected to 24 h glucose deprivation significantly doubled that of control cells (Figure 4B).

### Effects of AMPK and PKA coactivation on cell cycle progression and cell death during glucose starvation

**Figure 5:**
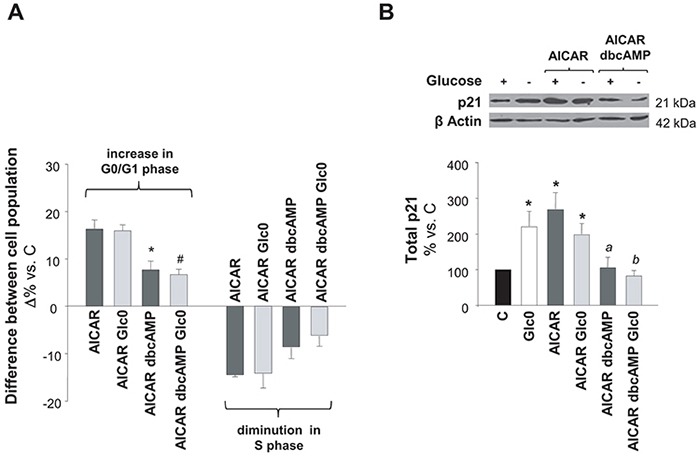
Effects of simultaneous AMPK and PKA activation on cell cycle progression C3A cells were incubated with 4.5 g/L glucose DMEM (C) or with no-glucose DMEM (Glc0), in the absence or presence of 100 μM dbcAMP (dbcAMP) or/and 1 mM AICAR (AICAR). **A.** Cells were fixed with cold 70% ethanol, marked with propidium iodide, and analyzed by flow cytometry. Bars show absolute differences in the percentages of cell populations in G0/G1 and S phases compared with C. *P<0.05 versus AICAR and *^#^P*<0.05 versus AICAR Glc0. **B.** Cell lysates were obtained and analyzed by western blotting to determine protein levels of p21. β Actin was used as loading control. *P<0.05 versus C. *^a^ P*<0.05 versus AICAR dbcAMP. *^b^ P*<0.05 versus AICAR dbcAMP Glc0. Values represent the mean ± SEM of 3 (A) or 4 (B) experiments.

AMPK and PKA signaling regulate diverse common metabolic pathways. Moreover, in adipocytes, PKA can inhibit lipolysis by negatively controlling AMPK activity [16, 17]. However, their overlap in the regulation of cell survival remains almost unexplored. According to our results, AMPK and PKA were simultaneously activated during glucose deprivation in C3A cells and the individual activation of each kinase led to cell cycle arrest and/or apoptosis. Hence, we further analyzed the result of PKA and AMPK coactivation on cell cycle and cell survival in cells subjected to glucose deprivation. As compared to cells treated with AICAR, cells treated with dbcAMP plus AICAR showed a significant diminution in cell cycle arrest, independently of the presence of glucose (Figure 5A). P21 is a central regulator of the cell cycle, which acts downstream AMPK and it is usually associated to AMPK modulation of cell cycle via the transcriptional activator p53 [21, 22]. As illustrated in Figure 5B, we demonstrated that the increase in p21 expression was significantly impaired when dbcAMP was added to AICAR treated C3A cells.

On the other hand, no significant changes in cell death were observed by simultaneous PKA and AMPK activation, when compared to each single treatment (data not shown). A simple explanation for this observation is that both kinases individually contributed to cell death (Figure 1C and 1D) but, at the same time, PKA prevented AMPK driven apoptosis, thus resulting in a non-cumulative increase of cell death in this situation of simultaneous of pharmacologic activation. In fact, either single activation of AMPK or PKA in fed cells led to similar increase in apoptosis.

Collectively, these results pointed out a putative counteraction of AMPK by PKA, which could limit the signaling effect of the former kinase as negative modulator of cell cycle progression and apoptotic death.

### PKA inhibits AMPK activation in hepatic cancer cells during glucose deprivation

**Figure 6:**
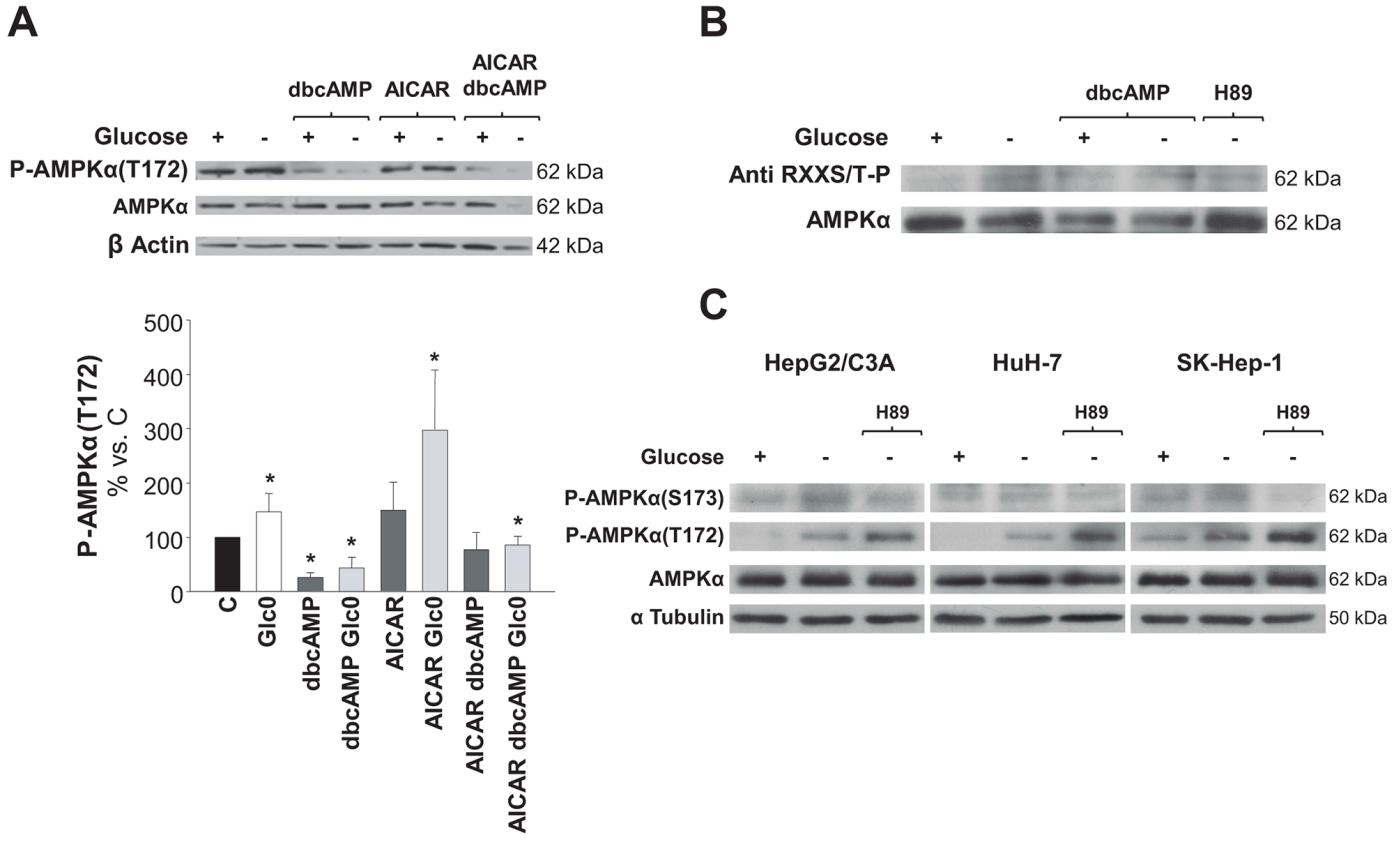
Regulation of AMPK activation by PKA phosphorylation in hepatic cancer cells C3A cells were incubated for 36 h (A) and 24 h (B) with 4.5 g/L glucose DMEM (C) or with no-glucose DMEM (Glc0). **A.** C and Glc0 cells were incubated in the absence or presence of 100 μM dbcAMP (dbcAMP) or/and 1 mM AICAR (AICAR). Cell lysates were obtained and analyzed by western blotting to determine protein levels of P-AMPKα(T172) and AMPKα. β Actin was used as loading control. Bars represent the mean ± SEM of 3 experiments. *P<0.05 versus C. **B.** In order to analyze the levels of AMPK that was phosphorylated by PKA, C and Glc0 cells were incubated with 100 μM dbcAMP (dbcAMP) or 5 μM H89 (H89). Four hundreds micrograms of cell lysates protein were subjected to immunoprecipitation with anti AMPKα, resuspended and loaded. PKA phosphorylated residues and total AMPKα levels were determined by western blotting. Band densities (mean values) of phosphorylated AMPKα relative to total AMPKα are 100, 160, 195, 179 and 115 Arbitrary Units in C, Glc0, C+dbcAMP, Glc0+dbcAMP and Glc0+H89, respectively. The blots are representative of 3 independent experiments. **C.** Specific phosphorylation of AMPKα(S173) was analyzed in C3A, HuH-7 and SK-Hep-1 cells incubated for 12 h with 4.5 g/L glucose DMEM, or with no-glucose DMEM with or without 5 μM H89 (H89). The same samples were also subjected to detection of P-AMPKα(T172) and AMPKα. α Tubulin was used as loading control. The blots are representative of 3 independent experiments.

To test the hypothesis emerged from our results, we first evaluated P-AMPKα(Thr172) levels in 36 h fed and fasted HepG2/C3A cells cultured in the presence of AICAR or/and dbcAMP. P-AMPKα(Thr172) levels were low whenever the cAMP analog was added to the culture medium. In fact, the increases in P-AMPKα(Thr172) levels (*P*<0.05) induced by either glucose deprivation, AICAR, or both were completely abrogated in the presence of dbcAMP (Figure 6A). In addition, maximum phosphorylation levels (about 20 fold the control levels) were achieved in cells treated with AICAR plus the PKA inhibitor H89 (data not shown).

We then considered detecting the amount of AMPKα phosphorylated by PKA in fed and fasted C3A cells. AMPKα possesses three bonafide sites which are phosphorylated by PKA: S173, S485/491 and S497 [20]. Immunoprecipitation of the catalytic subunit followed by immunodetection of phospho Ser/Thr residues by a specific antibody showed that AMPKα was barely phosphorylated by PKA in C3A cells in fed conditions. However, glucose deprivation entailed a clear increase in AMPKα phosphorylation by PKA. The fraction of AMPKα phosphorylated by PKA was also increased in the presence of dbcAMP, although this effect was not additive to the effect of glucose deprivation. Concomitantly, the increase in that AMPKα phosphorylated fraction observed after glucose fasting was prevented by the presence of the PKA inhibitor H89 (Figure 6B).

To investigate the putative phosphorylation of the PKA sensitive residue Ser173 of AMPKα, specific detection of phospho-AMPKα(Ser173) was performed in HepG2/C3A, HuH-7 cells and in SK-Hep-1 cells. During glucose deprivation the levels of phosphorylated AMPKα(Ser173) were significantly increased and this was blocked by the presence of the PKA inhibitor H89. Noteworthy, H89 enhanced the phosphorylation of Thr172 during glucose deprivation (Figure 6C).

### Abrogation of AMPKα phosphorylation in Ser173 generates a cell line more sensitive to cell death during glucose restriction

**Figure 7:**
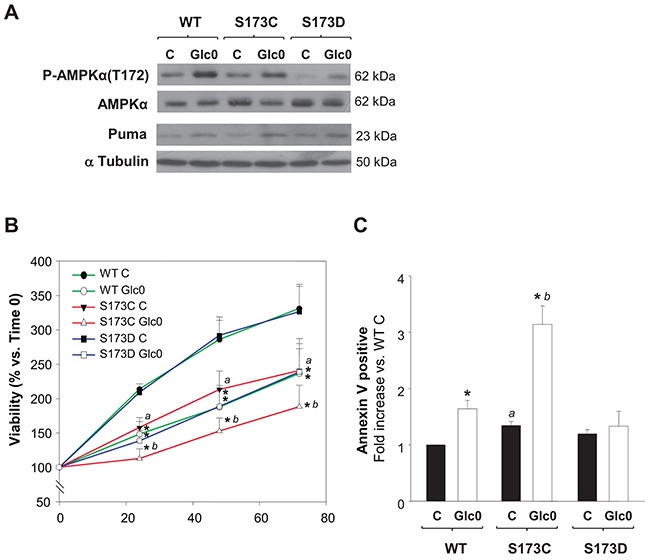
Disruption of AMPKα1(S173) increases cell death during glucose starvation in hepatocarcinoma derived cells C3A cells stably expressing either AMPKα1(S173C) (S173C), AMPKα1(S173D) (S173D) or wild type AMPKα1(S173) (WT) were incubated with 4.5 g/L glucose DMEM (C) or with no-glucose DMEM (Glc0). **A.** After 12 h of glucose deprivation cell lysates were obtained and analyzed by western blotting to determine protein levels of P-AMPKα(T172), AMPKα and Puma. α Tubulin was used as loading control. The blots in the picture are representative of 3 independent experiments. **B.** Cells attached to microplates were cultured for 0, 24, 48 and 72 h. MTT assay was performed as described in *Materials and Methods*. Results are presented as the percentage of the absorbance of the cells at 0 h. **C.** After 36 h cells were stained with Annexin V-FITC and propidium iodide (PI) and the percentages of apoptotic cells were determined by flow cytometry analysis. Bar charts represent fold increase respect to WT controls (WT C) in the proportion of cells undergoing apoptosis (Annexin V positive). WT C apoptotic cells (mean value): 9%. Values represent the mean ± SEM of 3 experiments. *P<0.05 versus C. *^a^ P*<0.05 versus WT C. *^b^ P*<0.05 versus WT Glc0.

In accordance with our results, AMPKα(S173) phosphorylation by PKA effectively prevents T172 phosphorylation in AMPKα activation site [16], a property also verified in tumor cells derived from HCC, HuH-7, by other authors [23]. Hence, we used a genetic approach to explore if PKA inhibitory effect on AMPK activity in our setting was achieved by AMPKα1(S173) phosphorylation. We constructed stable C3A cell lines expressing the phospho-mimetic AMPKα1(S173D) (S173D) or the unphosphorylable AMPKα1(S173C) (S173C) mutants and a cell line expressing the wild type AMPKα1 subunit (WT), under the same promoter. We first tested the impact of this regulation site by comparing the levels of activation of AMPK (P-AMPKα(Thr172)) in these clones in different conditions. As expected, twelve hours of glucose deprivation increased P-AMPKα(Thr172) levels in WT cells. Similarly, the levels of active AMPK were increased in S173C and S173D glucose starved cells. Nevertheless, P-AMPKα(Thr172) levels were drastically reduced both in fed and fasted S173D cells (Figure 7A). Thus, mimicking S173 phosphorylation with AMPKα1(S173D) mutation led to decreased T172 phosphorylation in C3A cells, confirming that S173 phosphorylation by PKA counterregulates AMPK activation in C3A cells. At the same time, we analyzed the expression of Puma, which is a well-known target of the AMPK-p53 transactivation pathway responsible of apoptotic induction in different cell lines. After 12 h of glucose deprivation, Puma levels were already increased either in WT, S173C or S173D cells. However, compared with the effect in WT cells, the induction (mean value) was partially prevented in the S173D (−50%), whereas, conversely, it was potentiated in S173C cells (+60%) (Figure 7A).

In turn, S173C, S173D and WT cells cultured with or without glucose were subjected to cell cycle and cell death analysis. No significant changes in glucose starving induced cell cycle arrest were evident among the clones (data not shown). The time dependent increase in the number of viable cells was reduced in the S173C cells exposed to both conditions, whilst S173D cells behavior was similar to that of WT cells (Figure 7B). On the other hand, the induction of apoptosis after glucose deprivation was avoided in S173D cells while, conversely, after 36 h-glucose deprivation, the percentage of apoptotic S173C cells doubled the percentage of apoptotic WT cells (*P*<0.05) (Figure 7C).

## DISCUSSION

Early studies in rat HCC derived cells show that overexpression of constitutively active AMPK leads to apoptosis [24]. Concomitantly, in cells derived from human HCC pharmacological activation of AMPK by different drugs entails either growth arrest or cell death [12–15]. In accordance with our own results in primary cultured hepatocytes [7], cAMP analogues commonly used as PKA activators also impair viability of HCC cells [9]. AMPK and PKA are implicated in the network of signaling pathways that control survival in diverse eukaryotic cells undergoing energy stress [25]. The present study provides strong evidence supporting the interaction of AMPK and PKA pathways in the decrease in survival of HCC cells during glucose starvation.

We first determined the changes in viability and in cell cycle in HCC cells subjected to glucose withdrawal. Twelve-hour glucose deprivation was enough to provoke a marked decrease in the number of viable cells. This was attributed to cell cycle arrest in G0/G1 and induction of both apoptotic and necrotic cell death. Each type of cell death has been previously described as the exclusive cause of cell death in glucose starved HepG2 cells [4, 6]. In our hands apoptosis and necrosis occurred simultaneously in HepG2/C3A cells subjected to this condition.

AMPK and PKA activities were monitored in glucose starved and fed cells and rapid activation of both kinases was detected following glucose withdrawal. Dissection of individual signaling was achieved by silencing the catalytic subunit of AMPK AMPKα1, and by inhibiting PKA activity. Furthermore, we studied the effect of AMPK and PKA coactivation compared to single activation. The results of these experiments support that: First, AMPK activation is the main responsible of cell cycle arrest after glucose restriction. Second, both AMPK and PKA participate in the pathways leading to the cell death associated to this nutritional stress in HCC cells as well as in liver cancer cells from non-hepatocitary origin.

AMPKα possesses different PKA and AKT phosphorylable sites. Among them, phosphorylation of AMPKα(S173), AMPKα(S485/491) and AMPKα(S497) by PKA have been postulated to interfere in the regulation of AMPK signaling [16, 20]. AMPKα(S173) phosphorylation by PKA allosterically impedes AMPKα(T172) phosphorylation and AMPK activation in adipocytes [16]. Regarding the phosphorylation of these sites in hepatic cells, different studies in HuH-7 cells demonstrate that AMPKα(S485/491) phosphorylation by AKT leads to AMPK inhibition [26], whereas, similarly to what is described in adipocytes, phosphorylation of AMPKα(S173) by PKA also inhibits AMPK activation [23]. The effect of this inhibition on the survival response of HCC cells has never been studied before. Here we demonstrated that AMPKα1 was phosphorylated by PKA in Ser173 in different liver cancer derived cells, what led to decreased AMPK activation, and that this occurred physiologically during glucose deprivation. Therefore, the cAMP-PKA axis arose as an inhibitor of AMPK antiproliferative effects in these tumor cells. In fact, during glucose restriction, HepG2/C3A cells stable expressing the unphosphorylable AMPKα1(S173C) mutant showed enhanced cell death, which was comparable with the effect of PKA inhibition in isogenic cells. Moreover, the increase in the levels of Puma elicited by glucose deprivation was negatively associated with the phosphorylation of AMPKα1 (S173). Puma is a pro-apoptotic BH3-only protein that acts upstream the pore formation in the mitochondrial outer membrane, which is upregulated by transcriptional activation via AMPK-p53 during energetic stress in different cancer cells [27]. In fact, Puma was early induced after glucose restriction and the extent of its induction could explain, at least in part, the different levels of apoptotic death observed in phosphomimetic and non-phosphorylable AMPKα1 (S173) mutants, respectively. Thus, AMPKα1 (S173) emerges as a PKA target residue that negatively controls the AMPK antitumoral effects in HCC cells subjected to glucose starvation.

In our setting, PKA and AMPK were simultaneously activated after glucose deprivation. We demonstrated that, in this scenario, PKA has a dual role: it favors an early diminution of cell viability, and, at a later time, it limits AMPK activation, thus decreasing apoptosis. This is consistent with the pleiotropic actions of PKA and its contrasting effects in different contexts. Activation of cAMP-PKA is postulated as an unequivocal antitumoral pathway in liquid cancers, inducing both cell cycle arrest and apoptosis [28, 29]. However, in HCC, as in other cancers, results are divergent. Whereas cAMP analogs increase cell cycle arrest and cell death in diverse HCC cell lines [9], the activation of cAMP-PKA axis induces malignant features in cells obtained from hepatoma of cirrhotic mice, [30]. In this cell line, promotion of survival is explained by the following hallmarks which were confirmed in HCC patients as well: 1. Constitutive activation of Ras-PKA pathway; 2. Aberrant hyperphosphorylation of LKB1 (Ser428) by PKA; 3. Blockage of AMPK activation [30].

Recent studies reveal that signaling kinases are recognized as potential therapeutic targets in liver cancer [31]. Among them, AMPK has emerged as a main player both in the development and treatment of HCC: On one hand, mRNA levels of the isoform 2 of the catalytic subunit, AMPKα2, are dramatically decreased in human HCC, whilst stable loss of AMPKα2 increases tumorigenity of HCC derived cells injected in nude mice [19]. On the other hand, AMPK activation –which is lower inside the HCC tumor than in the neighbor normal tissue– negatively correlates with the growth index and size of tumors [13, 29]; and activating AMPK with metformin or cannabinoids diminishes the growth of HCC xenografts [13, 15]. Our studies on cell cycle arrest and death in glucose-fasted HCC cells also point out the key role of AMPK in the HCC scenario, and suggest that glucose starvation alone or together with pharmacological AMPK activators can be targeted to impair tumor growth. In fact, dietary intervention in cancer is an attractive field, not yet enough studied in human patients [32, 33]. Fasting alone or combined with chemotherapy in mice bearing allografts of different human cancers, results in growth retard and increased apoptosis [34], which are associated to ROS production and other proapoptotic signaling pathways [35, 36]. In this connection, the putative antitumor effect of fasting-induced activation of AMPK in hepatocellular carcinoma deserves to be explored.

## MATERIALS AND METHODS

### Cell culture

Three different liver cancer cell lines were used throughout: C3A (HepG2/C3A, a clonal derivative of HepG2, ATCC, Manassas, VA) and HuH-7 (JCRB Cell Bank, Tokyo, Japan), both of them hepatocytic cells derived from HCC; and SK-Hep-1 (ATCC, Manassas, VA), mesenchymal cells derived from liver adenocarcinoma. Cells were grown on plastic dishes with 4.5 g/L glucose DMEM (Gibco, Thermo Fisher Scientific, Waltham, MA), supplemented with 10% fetal bovine serum and antibiotics. For glucose deprivation studies, cells were incubated in no-glucose DMEM (Gibco, Thermo Fisher Scientific) during the indicated periods. When indicated, the AMPK activator 5-Aminoimidazole-4-carboxamide ribonucleotide (1 mM, AICAR) (Cell Signalling Technology, Danvers, MA), the PKA inhibitor N-[2-(p-Bromocinnamylamino)ethyl]-5-isoquinolinesulfonamide dihydrochloride (5 μM, H89) (Santa Cruz Biotechnology Inc., Santa Cruz, CA) or the PKA activator dibutyryl-cAMP (100 μM, dbcAMP) (Santa Cruz Biotechnology Inc.) were added to the media.

### Reduction of AMPKα expression by RNA interference

In order to reduce AMPKα1 protein expression in hepatic cancer cells, specific 21 nucleotide double stranded RNA (siRNA) and a scrambled control were designed as we previously described [37], and synthesized using an Ambion commercial kit SilencerTMsiRNA (Thermo Fisher Scientific). The following sequence, chosen according the guidelines described by Elbashir et al. [38], which targets AMPKα1 nucleotides 1842-1864 was used: AACATTTCTGCATATTAGGCTCCTGTCTC. Cells were transfected using Dharmafect 4 reagent (Thermo Fisher Scientific). Experiments were performed 24 h after transfection, and the specific decrease in AMPKα expression was confirmed by immunoblotting.

### Generation of stable cell Lines (AMPKα1S173C, AMPKα1S173D and AMPKα1WT)

pCDNA3 plasmid harbouring Myc-AMPKα1(WT), Myc-AMPKα1(S173C) or Myc-AMPKα1(S173D) [39, 40], kindly given by Dr. Dietbert Neumann (Maastricht University, The Netherlands), were used to generate populations of C3A cells which stably express mutated forms of the AMPKα1(S173) residue. C3A cells were transfected by electroporation, as previously described [37]. After 24 h, the antibiotic Geneticin (500 μg/ml) (Invitrogen, Thermo Fisher Scientific) was added to the media in order to select the transfected cells. Clones expressing the AMPKα1 mutants were identified by immunodetection of c-Myc tag (c-Myc Antibody (9E10), Santa Cruz Biotechnology Inc.) and AMPKα (Cell Signaling Technology) protein expression, and grown in a medium containing Geneticin 200 μg/ml in conditions otherwise similar to parental cells.

### Cell viability and cell cycle progression studies

#### MTT assay

Cells were cultured in 96-well microplates and methylthiazolyldiphenyl-tetrazolium bromide (MTT, Sigma Chemical Co., St Louis, MO) was added into the culture medium at different time points to assess its metabolization, as we previously described [7]. After 2 h, cells were lysed by addition of DMSO and absorbance of the metabolite produced from viable cells was detected at 540 nm in a microplate reader (Beckman Coulter LD400). Results were expressed as percentage of absorbance in control cells.

#### Annexin V/propidium iodide assay

Cells were detached from the petri dishes by trypsinization in order to minimize cell re-aggregation and permit further cytometric studies, as we previously described for primary cultured hepatocytes [7], with slight modifications. After gently homogenization in the culture medium/PBS and harvest (5 min, 400 g), 100,000 cells were carefully re-suspended in the appropriate buffer. Apoptotic externalization of phosphatidylserine and cell death was assessed by staining with Annexin V-FITC and propidium iodide (Sigma Chemical Co.) coupled to flow cytometric analysis (Cell Sorter BD FACSAria II, BD Biosciences), following the manufacturers' instructions. Detection of green and red fluorescence was performed, and the proportion of Annexin V (apoptotic) and propidium iodide (necrotic –by the treatment and/or manipulation–) positive cells were determined in the indicated experimental groups. Green and red fluorescence intensities detected in non stained cells were used to set the thresholds for each channel.

#### Cell cycle analysis by flow cytometry

Cell distribution in the cell cycle was analyzed by determining the cellular DNA content by flow cytometry, as we previously described [41]. Briefly, 1 × 10^6^ cells were fixed with cold 70% ethanol and then washed with PBS and stained with 50 μg/ml propidium iodide (Sigma Chemical Co.) in a buffer containing 0.1% sodium citrate, 0.02 mg/ml RNAse, and 0.3% NP-40. Results were analyzed using WinMDi and Cylchred softwares.

### Preparation of subcellular fractions

To prepare cell lysates, cellular pellets were incubated for 30 min in RIPA buffer (1% Triton X-100 (v/v), 1% sodium deoxycholate (w/v), 0,1% SDS (w/v), 20 mM Tris, pH 8, 5 mM EDTA, 200 mM NaCl) supplemented with protease inhibitors (1 mM PMSF, 10 μg/mL leupeptin (Sigma Chemical Co.)) and phosphatase inhibitors (10 mM NaF, 2 mM Na_3_VO_4_, 100 nM calyculin A (Sigma Chemical Co.)) and sonicated.

Cytosolic fraction was prepared by differential centrifugation, as previously described [42]. Briefly, cells were washed with PBS, scrapped and collected by centrifugation at 1,000 g for 5 min. Cell pellets were re-suspended in isotonic STE buffer (0.25 M sucrose, 50 mM Tris–HCl (pH 7.4), 1 mM EDTA, and protease inhibitor mixture) and sonicated. Cell debris and nuclear fractions were removed by centrifugation and cytosolic and mitochondrial fractions separated by centrifugation at 16,000 g for 20 min.

Total protein concentrations of cell lysates and subcellular preparations were measured according to Lowry et al. [43].

### AMPK immunoprecipitation

After scrapping, cells were washed and lysed by the addition of lysis buffer (1% Triton X-100, 10% glycerol, 137 mM NaCl, 2 mM EDTA, 20 mM Tris-HCl), supplemented with protease and phosphatase inhibitors and sonicated.

Cell lysates (300-400 μg/sample) were incubated overnight with 1 μl of anti-AMPKα (Cell Signaling Technology) under constant agitation at 4°C. Four mg/mL of Protein A-Sepharose (Sigma Chemical Co.) was added and further incubation for 2-4 h was carried out. After that, samples were centrifuged 5 min at 5,000 r.p.m. Pellets were washed three times with PBS and finally the proteins bound to the Protein A-Sepharose were dissolved in 10 μl sample buffer and heated at 90°C for 10 min. The complete volumes of these recovered immunoprecipitates were loaded and subjected to immunoblot analysis.

### Immunoblotting

Cytosolic, total or immunoprecipitated proteins were separated by electrophoresis on SDS-polyacrylamide gels [44], and transferred to Immobilon polyvinylidene difluoride (PVDF) membranes (Perkin Elmer Life Sciences, Boston, MA, USA). Membranes were blocked with 5% non-fat milk/0.3% Tween/PBS, washed and incubated overnight with specific primary antibody [cytochrome c (Santa Cruz Biotechnology Inc.); α tubulin (Sigma Chemical Co.); β actin (Sigma Chemical Co.); phospho-(Ser/Thr) PKA Substrate (Cell Signaling Technology); p21 (Santa Cruz Biotechnology Inc.); AMPKα (Cell Signaling Technology); Phospho-AMPKα (Thr172) (Cell Signaling Technology); Phospho-AMPKα1/2 (Ser173) (Abcam, Cambridge, UK); Puma (Santa Cruz Biotechnology Inc.)]. Membranes were washed and probed with the appropriate secondary antibody conjugated to horseradish peroxidase. Bands were detected by chemiluminescence reaction (Amersham Pharmacia Biotech, Piscataway, NJ), after exposition to Kodak XAR film. Bands were quantified using the Image J software. In preparing the figures, brightness and contrast were adjusted in order to improve visualization.

### Statistical analysis

Data were expressed as mean ± SEM. Student t test was used for comparison between groups. *P* < 0.05 was considered statistically significant.

## Abbreviations

HCC: hepatocellular carcinoma
AMPK: AMP activated kinase
PKA: cAMP-protein kinase A
AICAR: 5-Aminoimidazole-4-carboxamide ribonucleotide
dbcAMP: dibutyryl-cAMP
PI: propidium iodide
siRNA: small interfering RNA
KD: knock down
ROS: radical oxygen species
WT: wild type
Puma: p53-upregulated modulator of apoptosis
DMEM: Dulbecco modified Eagle medium
